# Leptin as a key driver for organ fibrogenesis

**DOI:** 10.1126/sciadv.ady7904

**Published:** 2025-10-22

**Authors:** Xue-Nan Sun, Shiuhwei Chen, Shangang Zhao, Jan-Bernd Funcke, Megan Virostek, Line Pedersen, Chao Li, Chanmin Joung, Qian Lin, Yan Li, Ayanna Cobb, May-Yun Wang, Kyounghee Min, Lisandro Maya-Ramos, Giovanna Degasperi, Junquan Liu, Ningyan Zhang, Zhiqiang An, Diana R. Tomchick, R. Max Wynn, Da Young Oh, Philipp E. Scherer

**Affiliations:** ^1^ Touchstone Diabetes Center, University of Texas Southwestern Medical Center, Dallas, TX 75390, USA.; ^2^ Sam and Ann Barshop Institute for Longevity and Aging Studies, Division of Endocrinology, Department of Medicine, University of Texas Health Science Center at San Antonio, San Antonio, TX 78229, USA.; ^3^ Texas Therapeutics Institute, Brown Foundation Institute of Molecular Medicine, University of Texas Health Science Center at Houston, Houston, TX 77030, USA.; ^4^ Department of Biophysics, University of Texas Southwestern Medical Center, Dallas, TX 75390, USA.; ^5^ Department of Biochemistry, University of Texas Southwestern Medical Center, Dallas, TX 75390, USA.

## Abstract

Leptin, a hormone primarily secreted by adipocytes, regulates energy balance and systemic metabolism through its interaction with the leptin receptor (LEPR). Beyond these functions, leptin signaling has been implicated in the pathogenesis of tissue fibrosis. Here, we report the x-ray crystal structures of a leptin-neutralizing antibody (hLep3) in the unbound and leptin-bound states. The interaction of this antibody with leptin mimics the interaction of the LEPR with leptin, providing direct insights into the mechanism by which the antibody disrupts leptin signaling. We furthermore evaluate the therapeutic potential of neutralizing leptin with this antibody across distinct mouse models of fibrosis affecting the kidney, liver, lung, heart, and blood vessels. Leptin neutralization markedly inhibited fibrosis progression in all models. Mechanistically, suppression of leptin activity reduces pro-inflammatory and profibrotic processes, underscoring its therapeutic potential. These findings suggest that leptin signaling plays a vital role in tissue fibrosis and that treatment with a leptin-neutralizing antibody may be a promising therapeutic approach.

## Teaser

Neutralizing leptin reduces inflammation and fibrosis in mice, suggesting a potential treatment for fibrotic diseases.

## INTRODUCTION

Leptin, a hormone discovered in 1994, is predominantly synthesized and secreted by adipocytes (*1*, *2*). This pleiotropic hormone is renowned for its roles in regulating food intake and energy expenditure (*3*, *4*). However, individuals with obesity often exhibit elevated circulating leptin levels, suggesting severe leptin resistance. Recent studies demonstrate that controlled suppression of leptin activity, either genetically or pharmacologically, can improve metabolic health without leading to obesity (*5*, *6*).

Recent advancements in structural biology have shed much-needed light on the mechanism of leptin receptor (LEPR) activation. Cryo–electron microscopy (cryo-EM) studies have revealed the structure of a stabilized leptin:LEPR signaling complex that is characterized by a 3:3 stoichiometry, where each leptin binds to two distinct sites on adjacent receptor chains (*7*–*9*). These findings have not only elucidated the defects found in natural pathogenic and experimental antagonistic leptin variants (*10*, *11*) but also enabled the design of new biased LEPR agonists (*7*, *8*).

Leptin affects a wide range of physiological processes, including the regulation of fat mass, reproduction, immunity, hemostasis, angiogenesis, and blood pressure (BP). Leptin exerts its effects through binding to LEPR, which activates multiple signaling pathways (*12*). Among different LEPR isoforms, such as the soluble isoform (sLEPR), the short isoforms (e.g., LEPRa and LEPRc), and the long isoform (LEPRb), only the latter permits proper signaling via the Janus kinase 2 (JAK2)-signal transducer and activator of transcription 3/5 (STAT3/5) and phosphatidylinositol 3-kinase (PI3K)-protein kinase B (AKT) pathways (*13*, *14*). In contrast, the soluble and short isoforms appear to have specialized roles, such as regulating circulating leptin levels (*15*) or transporting leptin across the blood-brain barrier (*16*).

Fibrosis, characterized by excessive extracellular matrix (ECM) accumulation, leads to tissue dysfunction and is a major contributor to organ failure in chronic disease (*17*, *18*). This pathological process can affect multiple organs, including the kidney (*19*), liver (*20*), lung (*21*, *22*), and heart (*17*), severely impairing their respective functions. For these reasons, the development of effective antifibrotic therapies remains an urgent medical challenge.

Leptin’s role in fibrosis has gained increasing attention (*23*, *24*). Leptin has been described to promote fibroblast activation, proliferation, and ECM production, directly contributing to fibrotic processes (*25*). The JAK2-STAT3/5 and PI3K-AKT pathways, which can be activated by leptin engaging LEPRb, are well-known regulators of inflammation and fibrosis (*12*).

Despite the growing evidence of leptin’s contribution to fibrosis, the underlying mechanisms remain underexplored. Prior studies have suggested that neutralizing leptin or blocking LEPRb signaling may mitigate fibrotic responses (*5*). However, a comprehensive evaluation of the potential of leptin neutralization across diverse models of fibrosis is still lacking.

This study aims to address this gap by systematically evaluating the effects of suppressing leptin activity with a monoclonal leptin-neutralizing antibody in multiple mouse models of fibrosis, specifically of the kidney, liver, lung, and blood vessels. We hypothesized that neutralizing leptin could attenuate fibrotic responses and improve tissue function in these models. By delineating how altered leptin:LEPR signaling may contribute to fibrosis, we hope to pave the way for novel therapeutic strategies that target leptin activity to benefit patients suffering from fibrotic conditions.

## RESULTS

### Identification and characterization of a leptin-neutralizing antibody

We previously described the identification of a human monoclonal immunoglobulin G (IgG) antibody (hLep3; in the following, simply referred to as “LepAb”) capable of neutralizing human leptin as well as mouse leptin (*5*). However, the structural basis of how LepAb neutralizes leptin is unknown, which hampers its application in the clinic. We therefore aimed to determine the structure of the LepAb:leptin complex.

**Fig. 1.**
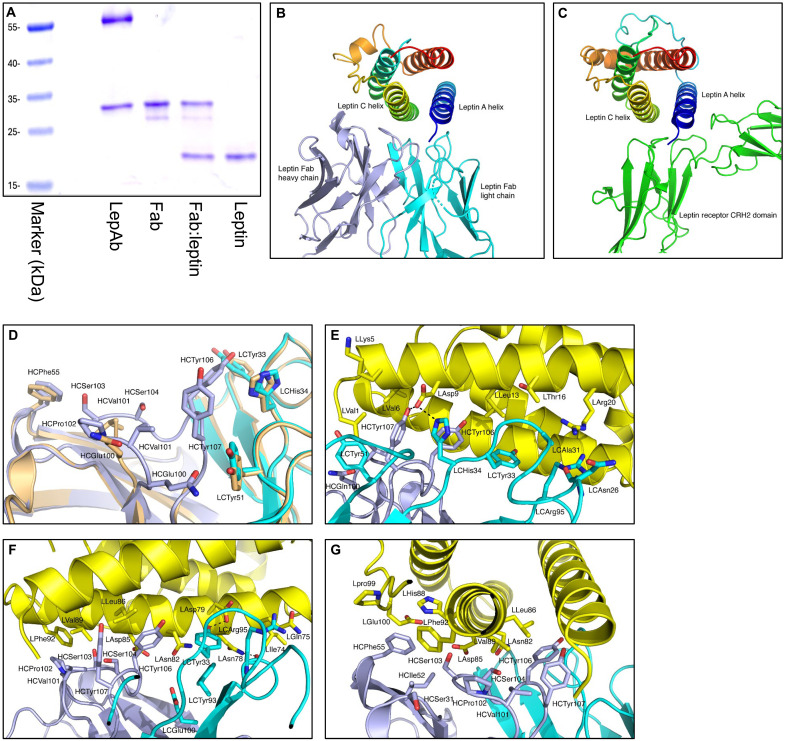
Structural characterization of a leptin-neutralizing antibody. (**A**) SDS-PAGE gel of a leptin-neutralizing antibody, Fab, Fab: leptin complex, and leptin under reducing conditions. (**B** and **C**) The interaction of the Fab with leptin mimics the LEPR CRH2 domain, which predominantly binds helix A of leptin, while the Fab also interacts with both helices A and C and the loop between helices C and D of leptin. (**D**) The superposition of the unbound Fab (orange) and Fab:leptin complex (light chain, cyan; heavy chain, light blue) shows conformational changes, with a root mean square deviation of 0.44 Å for 220 Cα atoms. The largest difference occurs in the CDR H3 loop (heavy chain residues 100 to 107), where residues 102 to 107 are unmodeled in the unbound Fab because of a lack of interpretable electron density. (**E**) Fab heavy and light chains interact with conserved residues on leptin helix A (yellow), identified by CCP4’s Ncont (≤5.0 Å) (*72*). Fab light and heavy chains are shown in cyan and light blue, respectively. Residues are labeled L (leptin), HC (heavy), and LC (light). Hydrogen bonds are depicted as dashed black lines. (**F**) Fab heavy and light chains interact with conserved residues on leptin’s helix C. For clarity, selected interactions from (D) are not shown. Hydrogen bonds are depicted as dashed black lines. (**G**) Residues from the Fab heavy chain interact with conserved residues of the leptin’s helix C and the loop between helices C and D. The charged carboxylate of LGlu^100^ points away from the hydrophobic cluster of HCPhe^55^, LHis^88^, and LPhe^92^. For clarity, selected interactions from (C) are not shown. Leptin helices A and C and the residues from the loop between helices C and D contribute to all the interactions with the heavy and light chains of the neutralizing antibody.

Using papain digestion, we prepared a Fab from the LepAb, which we subjected to crystallization either in complex with human leptin or alone. Reducing SDS–polyacrylamide gel electrophoresis (SDS-PAGE) analysis confirmed the successful purification of the Fab and the Fab:leptin complex with distinct bands corresponding to each component (Fig. 1A). Using x-ray crystallography, we solved the structure of the Fab:leptin complex [Protein Data Bank (PDB) ID: 9PUO] as well as the isolated unbound Fab (PDB ID: 9PUK).

While the Fab:leptin interaction (Fig. 1B and fig. S1A) overall mimics that of the LEPR cytokine receptor homology 2 (CRH2) domain with leptin (Fig. 1C and fig. S1B), we observed several noteworthy differences. The LEPR CRH2 domain forms more hydrogen bonds with leptin than the Fab does (five versus three), yet the Fab epitope involves a broader region, including leptin’s helix C and residues from the C-D loop. This interaction bears greater similarity to the binding geometry of interleukin-4 (IL-4) to its receptor (*26*, *27*), with comparable buried surface areas [Fab:leptin, 870 Å^2^; IL4R (IL-4 receptor):IL-4, 810 Å^2^] (fig. S1C). Notably, we observe only minor conformational changes in the Fab upon complex formation (Fig. 1D). In the Fab:leptin complex, residues of leptin helix A interact with residues from both the Fab light chain (Asn^26^, Ala^31^, Gly^32^, Tyr^33^, His^34^, Tyr^51^, and Arg^95^) and the Fab heavy chain (Gln^100^, Val^101^, Tyr^106^, and Tyr^107^) (Fig. 1E). Key interactions include a hydrogen bond between the Fab heavy chain Tyr^107^ and leptin Asp^9^ and a salt bridge between the Fab light chain His^34^ and leptin Asp^9^. Similarly, leptin helix C engages with residues from the Fab heavy chain (Ser^31^, Ile^52^, Phe^55^, Val^101^, Pro^102^, Ser^103^, Ser^104^, Tyr^106^, and Tyr^107^) and Fab light chain (Tyr^33^, Arg^95^, Gly^98^, and Glu^100^) (Fig. 1, F and G). Crucial interactions include hydrogen bonds between Fab light chain Tyr^33^ and leptin Asp^79^ and between Fab light chain Arg^95^ and leptin Asn^78^. Two residues from leptin’s C-D loop (Pro^99^ and Glu^100^) further stabilize the Fab heavy chain interaction. The observed epitope overlaps between the Fab:leptin and LEPR CRH2:leptin complexes offer valuable insights into the antibody’s mechanism of action (Fig. 1, D and G). Several conserved residues are critical for binding specificity, underscoring their significance for leptin’s interactions with the LEPR as well as LepAb (Fig. 1 and Table 1).

**Table 1.** Interaction analysis of leptin residues in leptin:Fab and leptin:LepR structures. List of leptin residues that interact (within 5.0 Å of non-H atoms) with residues in the leptin:Fab and leptin:leptin receptor (LepR) structures (PDB ID: 8x80), as determined in the program AreaIMol in the CCP4 program suite. Asterisks denote hydrogen bonds and/or salt bridges. Numbers for leptin residues in parentheses denote the numbering used in the leptin:LepR structure. Leptin residues in bold are highly conserved.

Leptin	Fab heavy chain	Fab light chain	LepR CRH2 domain
**V1 (22)**	Q100		Y472
	V101		
	Y107		
**P2 (23)**		Y51	
**K5 (26)**	Y107		Y472
**V6 (27)**	Y107		Y472
**D9 (30)**	Y106	H34*	Y472*
	Y107*		L506
			D532
**T12 (33)**			E565
			N566
**L13 (34)**	Y106	Y33	L505
			L506
**K15 (36)**			E565
**T16 (37)**		A31	L442
		G32	L505
			F563
			E565
**T19 (40)**			V562
			F563
			E565
**R20 (41)**		N26	Y441
		A31	Y441
		R95	L442*
			T443
			I503
			L505
			F563
**R71 (92)**			T443
			Q501*
			P502
**I74 (95)**		R95	
**Q75 (96)**		R95	L442
			Q501
			P502
			I503*
N78 (99)		Y33	P502
		Y93	I503
		R95	F504
		G98	
**D79 (100)**		Y33*	I503
		Y93	L505
		R95	
		E100	
**E81 (102)**			R468
			S470
			F504
			S507
**N82 (103)**	Y106		S470
			F504
			L505
			L506
			S507*
**D85 (106)**	I52		S470
	S103		
	S104		
**L86 (107)**	Y106		L471
	Y107		Y472
**H88 (109)**	F55		
	S103		
V89 (110)	V101		
	P102		
	S103		
F92 (113)	S31		
	F55		
	P102		
	S103		
**P99 (120)**	F55		
E100 (121)	F55		

To evaluate the functional consequences of LepAb binding to leptin, we cotransfected human embryonic kidney (HEK) 293 cells with LEPRb and a phosphorylated STAT3 (p-STAT3) luciferase reporter encoding plasmids and treated them with leptin in the presence of either LepAb or a control IgG antibody. This assay revealed that LepAb effectively inhibited leptin-induced STAT3 activation compared to the IgG control (fig. S1D). Together, our structural and functional analyses demonstrate that LepAb neutralizes leptin by preventing it from binding to and thus also activating its receptor.

### Assessment of local leptin signaling in mouse models of liver and kidney fibrosis

**Fig. 2.**
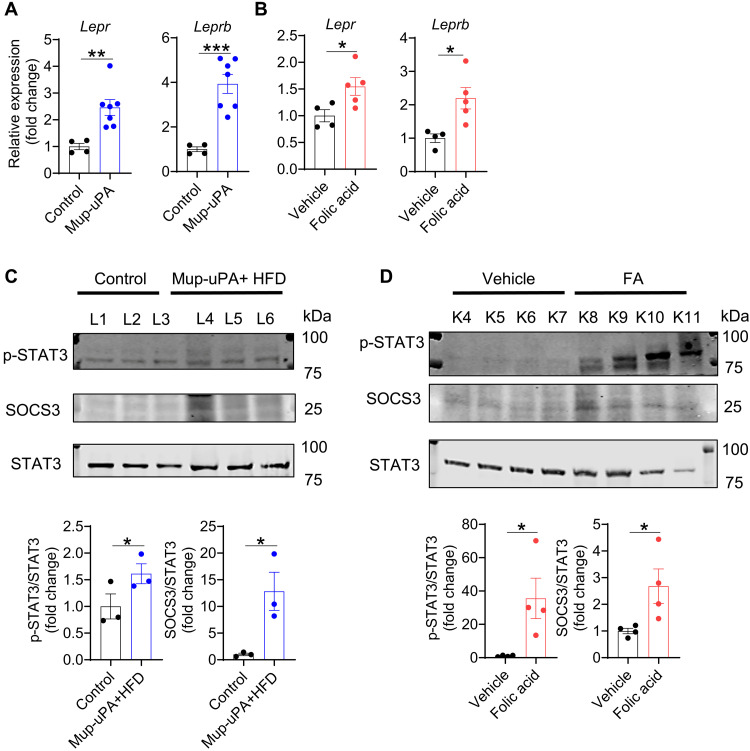
Local leptin signaling is elevated in liver and kidney fibrosis. For liver fibrosis induction, Mup-uPA (MU) mice and control littermates were fed an HFD for 12 weeks and then euthanized. For kidney fibrosis induction, wild-type mice were injected with folic acid (FA; 200 mg/kg) or vehicle and euthanized at day 7. (**A** and **B**) RT-qPCR analysis of total and long-isoform LEPR (*Lepr* and *Leprb*, respectively) mRNA expression in livers (A) (*n* = 4 to 7 per group) and kidneys (B) (*n* = 4 to 6 per group). (**C** and **D**) Immunoblot analysis of p-STAT3, STAT3, and SOCS3 protein expression in livers (C) (*n* = 3 per group) and kidneys (D) (*n* = 4 per group). Data are presented as the means ± SEM and were analyzed by a one-tailed Student’s *t* test. *P < 0.05; **P < 0.01; ***P < 0.001.

Most of leptin’s effects are mediated through LEPRb expressed in both the brain and peripheral tissues (*28*, *29*). To investigate leptin’s role in fibrogenesis, we first assessed the total and long-isoform LEPR mRNA expression in two distinct fibrosis settings, namely a model of liver fibrosis [major urinary protein (Mup)-urokinase-type plasminogen activator (uPA) mice fed a high-fat diet (HFD)] and a model of kidney fibrosis (mice treated with a bolus of folic acid). Intriguingly, following fibrosis induction, the total and long-isoform LEPR mRNA expression was strongly up-regulated in the liver and kidney of these mice (Fig. 2, A and B). We subsequently assessed surrogates of leptin signaling by analyzing the protein levels of p-STAT3, total STAT3, and suppressor of cytokine signaling 3 (SOCS3) in the fibrotic tissues. Immunoblotting revealed markedly elevated p-STAT3 and SOCS3 protein levels in the livers of HFD-fed Mup-uPA mice (Fig. 2C) and elevated p-STAT3 and SOCS3 protein levels in the kidneys of folic acid–treated mice (Fig. 2D). These findings suggest that fibrogenesis may be accompanied by elevated local leptin signaling, raising the possibility that leptin could serve as a potential target for fibrosis treatment.

### Leptin neutralization diminishes kidney fibrosis in folic acid–treated mice

**Fig. 3.**
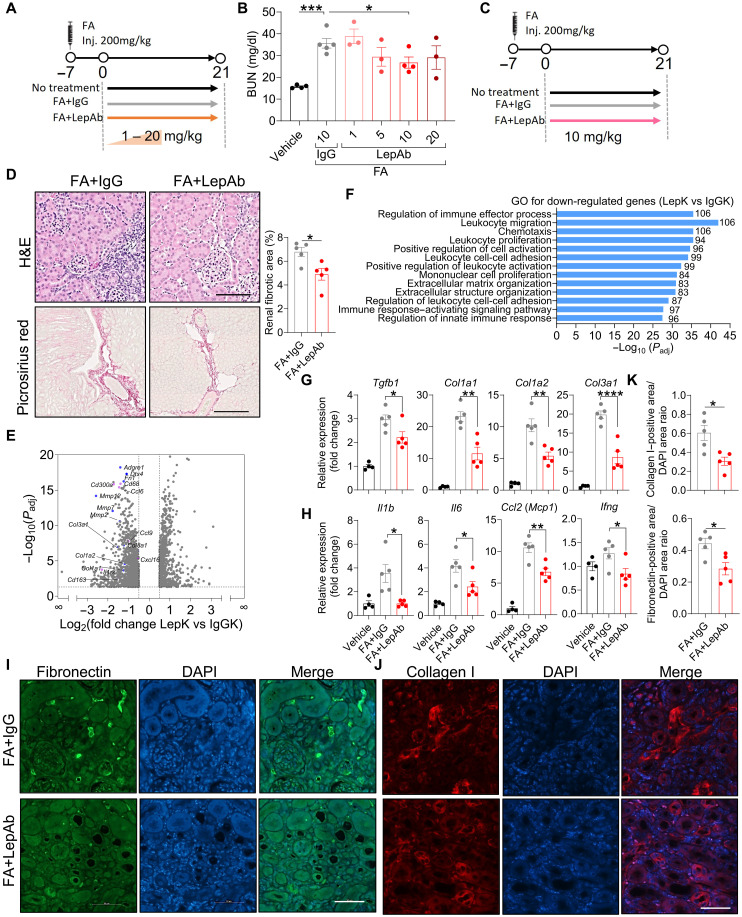
Leptin neutralization diminishes kidney fibrosis. (**A**) Experimental setup of folic acid–induced kidney fibrosis. (**B**) Serum BUN levels. (**C**) Schematic for mice that were subjected to LepAb treatment following folic acid–mediated acute kidney injury. (**D**) H&E and picrosirius red staining of kidney. Representative microphotographs are shown (*n* = 5 per group). Scale bars equal 125 μm (H&E staining) and 250 μm (Picrosirius red staining). (**E**) Volcano plots display the fold change (*x* axis) versus adjusted *P* value (*y* axis) of transcriptomic data from kidneys of IgG-treated (IgGK) versus leptin antibody–treated (LepK) mice. Mean values from *n* = 3 per group. (**F**) Labels identify gene clusters showing enrichment GO analyses for LepK versus IgGK. (**G** and **H**) RT-qPCR analysis of fibrotic (G) and inflammatory (H) gene mRNA expression in the kidney (*n* = 4 or 5 per group). DAPI, 4′,6-diamidino-2-phenylindole. (**I** and **J**) Immunofluorescence staining of fibronectin (FN) and collagen I (COL1) of the kidney. Representative microphotographs are shown (*n* = 5 per group). The scale bar equals 50 μm. (**K**) Quantification of immunofluorescence staining for FN (I) and COL1 (J) in the kidney. [(C), (D), (G), (H), and (K)] Data are presented as the means ± SEM and were analyzed by a two-tailed Student’s *t* test or one-way ANOVA with Dunnett’s test. *P < 0.05; **P < 0.01; ***P < 0.001; ****P < 0.0001.

Given the potential up-regulation of local leptin signaling following folic acid–induced acute kidney injury, we assessed the therapeutic potential of leptin neutralization in this context. Mice were treated with increasing doses of LepAb (1 to 20 mg/kg) or control IgG, and fibrosis outcomes were evaluated (Fig. 3A). The body weight remained stable across all groups (fig. S2A), and LepAb treatment modestly attenuated the reduction in kidney size typically observed after folic acid injection (fig. S2B). Biochemical analysis showed a dose-dependent reduction in blood urea nitrogen (BUN) and serum creatinine levels with LepAb administration, indicative of improved renal function (Fig. 3B and fig. S2D). Similarly, the mRNA expression of several fibrotic markers decreased in a dose-dependent manner (fig. S2E). As there was no further improvement between the 10 and 20 mg/kg doses, the 10 mg/kg dose was selected for subsequent studies (Fig. 3C). Histological examination confirmed that LepAb treatment at 10 mg/kg reduced ECM accumulation and improved renal architecture compared to IgG controls (Fig. 3D). To further investigate underlying mechanisms, we performed RNA sequencing (RNA-seq) (GSE303498) on kidneys from mice treated with LepAb or IgG following vehicle- or folic acid–induced injury. Differential expression and Gene Ontology (GO) analyses revealed that genes associated with collagen production and inflammation were notably up-regulated in the IgG-treated injured kidneys (IgGK) compared to vehicle-treated controls (WTK), whereas these same gene programs were markedly suppressed in LepAb-treated kidneys (LepK) (fig. S2, F and G, and Fig. 3, E and F). Reverse transcription quantitative polymerase chain reaction (RT-qPCR) analysis validated these findings, showing meaningfully reduced expression of fibrogenic genes (*Tgfb1*, *Col1a1*, *Col1a2*, and *Col3a1*) and inflammatory mediators (*Ccl2*, *Il1b*, *Il6*, and *Ifng*) in LepAb-treated kidneys (Fig. 3, G and H). These transcriptomic and molecular data were corroborated by immunofluorescence staining, which demonstrated reduced deposition of fibronectin (FN) and collagen I (COL1) in LepAb-treated mice relative to IgG controls (Fig. 3, I and K). Together, these findings indicate that leptin neutralization mitigates kidney fibrosis and improves renal function following acute injury by suppressing profibrotic and pro-inflammatory pathways.

### Leptin neutralization attenuates liver fibrosis in Mup-uPA mice fed an HFD

**Fig. 4.**
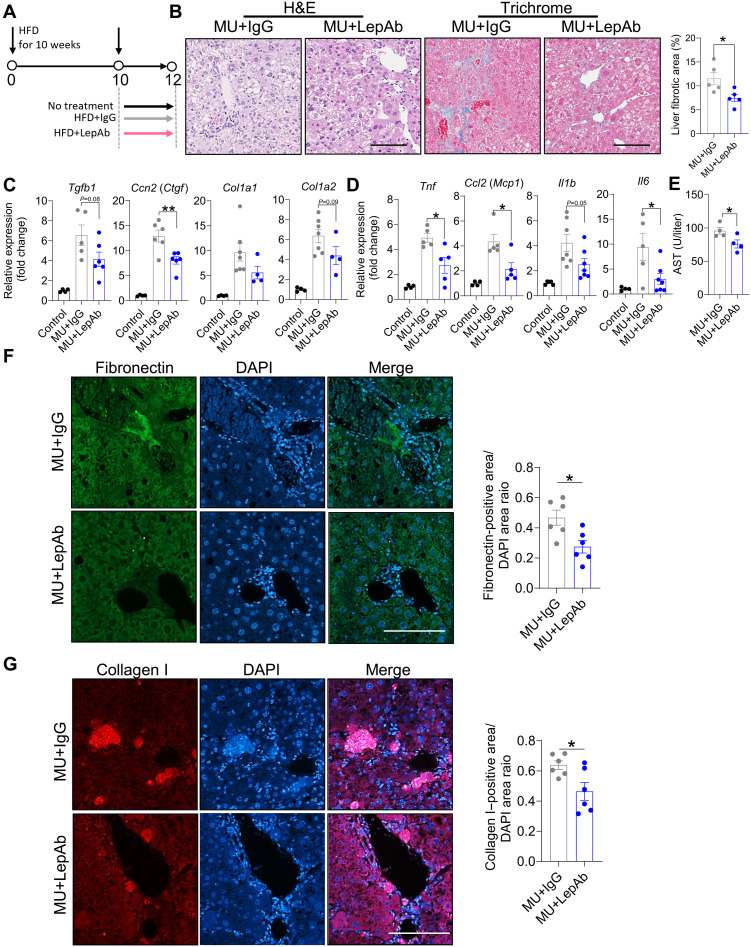
Leptin neutralization attenuates liver fibrosis. (**A**) Experimental setup of Mup-uPA– and HFD-induced liver fibrosis. (**B**) H&E and Masson’s trichome staining of the liver. Representative microphotographs are shown (*n* = 5 per group). The scale bar equals 125 μm. (**C** and **D**) RT-qPCR analysis of fibrotic (C) and inflammatory gene (D) mRNA expression in the liver (*n* = 4 to 7 per group). (**E**) Serum AST (*n* = 4 per group). (**F** and **G**) Immunofluorescence staining of FN and COL1 of the liver. Representative microphotographs are shown (*n* = 5 per group). The scale bar equals 100 μm. [(C) to (G)] Data are presented as the means ± SEM and were analyzed by a two-tailed Student’s *t* test or one-way ANOVA with Dunnett’s test. *P < 0.05; **P < 0.01.

Nonalcoholic steatohepatitis, now more commonly referred to as metabolic dysfunction–associated steatohepatitis (MASH), considerably increases the risk of hepatocellular carcinoma (HCC), with up to 50% of newly occuring HCC cases arising independently of viral infections (*30*). Obesity-related MASH can progress to liver fibrosis, cirrhosis, and eventually HCC (*31*–*33*). Our previous findings indicated that leptin signaling is increased under conditions of HFD-induced liver fibrosis in Mup-uPA transgenic mice. To evaluate the effects of leptin neutralization on MASH progression, Mup-uPA mice were fed an HFD for 10 weeks and then treated with LepAb or IgG control for an additional 2 weeks (Fig. 4A). Histological analysis showed that LepAb treatment effectively reduced hepatocyte ballooning and ECM deposition in the liver (Fig. 4B). Consistently, RT-qPCR analysis revealed noticeable reductions in the mRNA expression of key fibrotic and inflammatory markers (Fig. 4, C and D). Moreover, LepAb treatment improved liver function, as demonstrated by lower serum levels of aspartate aminotransferase (AST) (Fig. 4E), although alanine aminotransferase (ALT) levels were unaffected (fig. S3A). Immunofluorescence staining corroborated these results, showing decreased fibronectin and collagen I deposition in the liver of LepAb-treated mice (Fig. 4, F and G). Notably, body weights were not affected by either LepAb or IgG treatment, indicating that the observed therapeutic effects were independent of body weight changes (fig. S3B). These findings underscore the importance of leptin signaling for MASH pathogenesis and suggest that neutralizing leptin may help attenuate obesity-associated liver fibrosis.

### Leptin neutralization alleviates lung fibrosis in bleomycin-treated mice

**Fig. 5.**
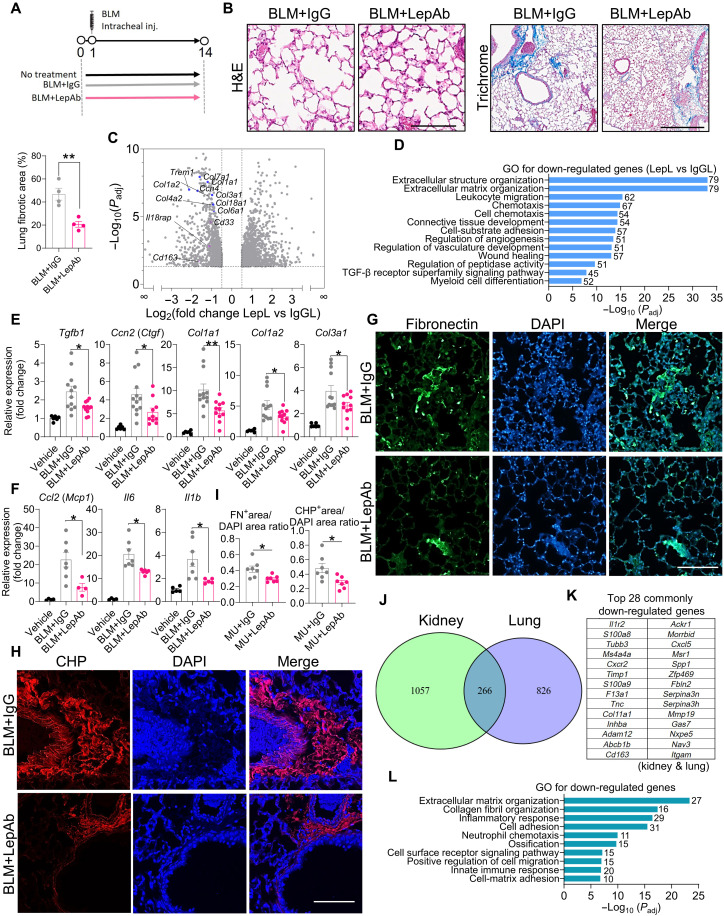
Leptin neutralization alleviates lung fibrosis. (**A**) Experimental setup of bleomycin (BLM)–induced lung fibrosis. (**B**) H&E and Masson’s trichome staining of lungs. Representative microphotographs are shown (*n* = 4 per group). For H&E staining, the scale bar equals 125 μm. For trichrome staining, the scale bar equals 500 μm. (**C**) Volcano plots display the fold change (*x* axis) versus adjusted *P* value (*y* axis) of transcriptomic data from mice treated with bleomycin or vehicle, followed by IgG (IgGL) or leptin antibody (LepL) administration. Mean values from *n* = 3 mice per group. (**D**) Gene clusters identified from hierarchical clustering, with selected clusters annotated by GO term enrichment analysis comparing IgG-treated (IgGL) and leptin antibody–treated (LepL) groups. (**E**) RT-qPCR analysis of fibrotic gene mRNA expression in the lung (*n* = 6 to 12 per group). (**F**) RT-qPCR analysis of inflammatory gene mRNA expression in the lung (*n* = 4 to 7 per group). (**G** to **I**) Immunofluorescence staining of FN (E) and CHP (F) of the lung and quantification of immunofluorescence staining of FN and CHP in the lung (I). Representative microphotographs are shown (*n* = 7 per group). The scale bar equals 100 μm. (**J**) Venn diagram showing the overlap of down-regulated genes in kidney and lung tissues after LepAb treatment. (**K**) Top 28 overlapping down-regulated genes identified on the basis of log_2_ fold change and adjusted *P* value common to the kidney and lung. (**L**) GO enrichment analysis of 266 shared down-regulated genes. Data are presented as the means ± SEM and were analyzed by a one-way ANOVA with Dunnett’s test or a two-tailed Student’s *t* test. *P < 0.05; **P < 0.01. FN^+^, FN-positive; CHP^+^, CHP-positive.

Pulmonary fibrosis is a progressive and debilitating condition, where timely intervention is critical to prevent irreversible lung damage (*34*). To assess the therapeutic potential of leptin neutralization in this context, we treated wild-type mice with bleomycin to induce lung fibrosis and administered either a LepAb or control IgG (Fig. 5A). Histological analysis revealed that LepAb treatment preserved alveolar architecture and reduced ECM deposition compared to controls (Fig. 5B). To understand the molecular basis for these improvements, we performed RNA-seq (GSE303498) on lung tissues from bleomycin- or vehicle-treated mice receiving either LepAb (LepL) or IgG (IgGL). Differential expression and GO analyses revealed that leptin neutralization notably reduced the expression of inflammation- and fibrosis-related gene programs (Fig. 5, C and D). These transcriptomic findings were corroborated by RT-qPCR, which showed reduced mRNA expression of key fibrogenic genes (*Tgfb1*, *Ccn2*, *Col1a1*, *Col1a2*, and *Col3a1*) and inflammatory cytokines (*Ccl2*, *Il1b*, and *Il6*) in the LepAb-treated group (Fig. 5, E and F). Consistent with these results, immunofluorescence staining demonstrated decreased deposition of fibronectin and a reduced signal from collagen-hybridizing peptide (CHP), a marker of ECM remodeling, in the lungs of LepAb-treated mice (Fig. 5, G and H). Together, these data indicate that leptin neutralization leads to reduced fibrotic remodeling, highlighting its potential as a therapeutic strategy for the treatment of pulmonary fibrosis.

To assess whether similar transcriptional changes occur in other organs, we compared down-regulated genes in the kidney and lung using a two-way Venn diagram. The analysis identified a total of 1323 down-regulated genes in the kidney and 1092 in the lung, with 266 genes overlapping, indicating a shared transcriptional response to LepAb treatment under fibrotic conditions. On the basis of log_2_ fold change and adjusted *P* values, the top 28 overlapping down-regulated genes are listed in Fig. 5K. GO analysis revealed that leptin neutralization may suppress collagen-related and inflammatory pathways in both lung and kidney fibrotic models (Fig. 5, J to L).

### Leptin neutralization mitigates aorta and kidney fibrosis but not heart fibrosis in angiotensin II–treated mice

**Fig. 6.**
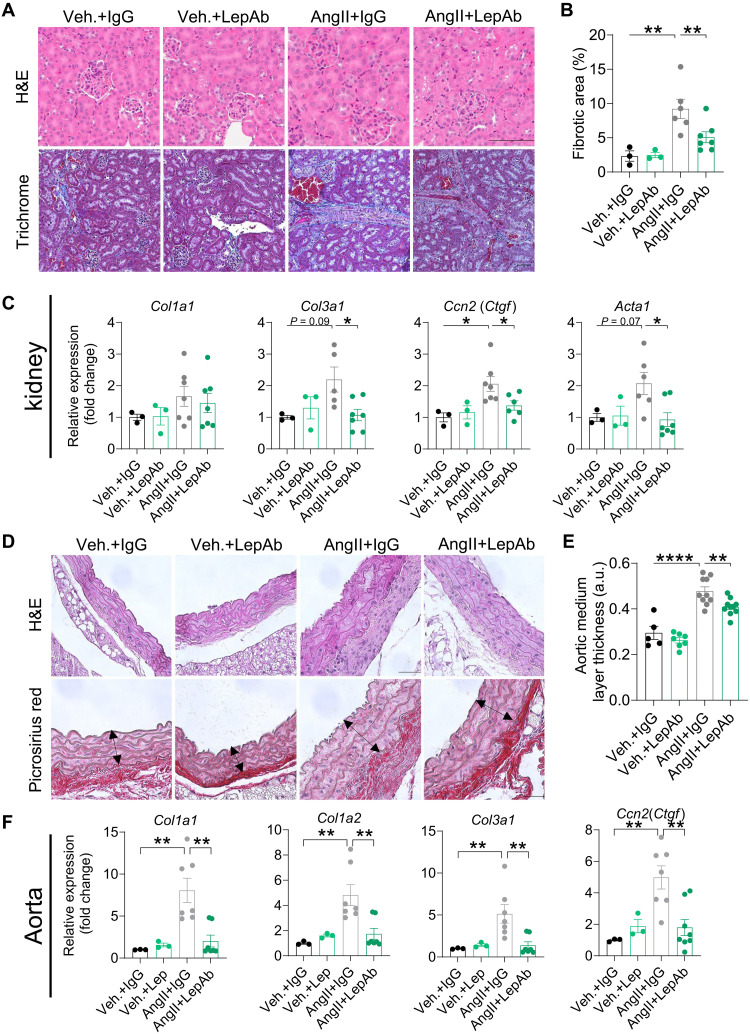
Leptin neutralization mitigates aorta and kidney fibrosis but not heart fibrosis. (**A** and **B**) H&E and Masson’s trichome staining of the kidney. Representative microphotographs (A) and quantification (B) are shown (*n* = 3 to 5 mice per group; 3 or 4 sections per mouse for quantification). For H&E staining, the scale bar equals 100 μm. For Masson’s trichome staining, the scale bar equals 50 μm (**C**) RT-qPCR analysis of fibrotic gene mRNA expression in the kidney (*n* = 3 to 6 per group). (**D** and **E**) H&E and Masson’s trichome staining of the kidney. Representative microphotographs (D) and quantification (E) are shown (*n* = 3 to 5 mice per group; 3 or 4 sections per mouse for quantification). The scale bar equals 50 μm. a.u., arbitrary units. (**F**) RT-qPCR analysis of fibrotic gene mRNA expression in the aorta (*n* = 3 to 5 mice per group). The scale bar equals 50 μm. Data are presented as the means ± SEM and were analyzed by a one-way ANOVA. *P < 0.05; **P < 0.01; **** *P* < 0.0001.

Leptin has been reported to contribute to the elevation of BP in obesity, but its effects on nonobese populations are less clear (*4*, *35*–*38*). To assess the impact of leptin neutralization on hypertension-driven organ fibrosis, we infused lean wild-type mice with angiotensin II (AngII) and treated them with either LepAb or control IgG for 14 days (fig. S4A). Tail-cuff BP measurements showed no substantial differences in systolic or diastolic BP between LepAb- and IgG-treated mice, independent of whether they were infused with AngII to induce hypertension or saline as a control (fig. S4, B and C). Similarly, there were no meaningful changes in the ventricular weight–to–body weight ratio between the treatment groups following AngII infusion (fig. S4D). Histological analysis of left ventricular sections revealed no changes in cardiac morphology or fibrosis between groups (fig. S4E). In line with these findings, the mRNA expression levels of fibrosis-related genes, including *Col1a1*, *Col1a2*, *Col3a1*, and *Ccn2* (*Ctgf*) in the heart were comparable between LepAb- and IgG-treated groups following AngII infusion (fig. S4F). In contrast, LepAb treatment effectively reduced kidney fibrosis in AngII-infused mice, shown by hematoxylin and eosin (H&E) and trichrome staining (Fig. 6, A and B). The mRNA expression of fibrosis-related genes, including *Col1a1*, Col*3a1*, *Ccn2* (*Ctgf*), and *Acta1*, in the kidney was attenuated after LepAb treatment (Fig. 6C). LepAb treatment also effectively decreased aortic medium thickness and fibrosis, as suggested by H&E and picrosirius red staining (Fig. 6, D and E). AngII-induced fibrotic genes of aortas were reduced in the LepAb-treated group compared to the control group (Fig. 6F). These findings suggest that while leptin neutralization does not affect hypertension-induced cardiac fibrosis, it does result in an attenuation of hypertension-induced kidney and aorta fibrosis, indicating that leptin signaling plays a crucial role in the fibrotic processes in these tissues.

## DISCUSSION

Leptin regulates systemic metabolism and tissue homeostasis by influencing energy balance, glucose and lipid metabolism, and immune responses (*39*–*42*). Beyond these roles, leptin has been implicated in the regulation of inflammation and fibrosis in peripheral organs, including the heart, kidneys, and liver (*12*). These pleiotropic effects of leptin are predominantly mediated through the long-form LEPR (LEPRb). Suppressing leptin signaling with either leptin- or LEPRb-neutralizing antibodies may therefore present a promising therapeutic strategy for fibrotic diseases.

In this study, we used x-ray crystallography to determine the structure of a potent leptin-neutralizing antibody as an unbound Fab as well as in complex with leptin. Our structural analyses provide critical insights into the mechanistic basis of this antibody’s actions and further deepen our understanding about how leptin engages its receptor. The resolution of the Fab:leptin complex revealed that electrostatic interactions, shape complementarity, and minor conformational changes contribute to antigen binding. Consistent with other antibody:antigen complexes, the Fab antibody undergoes limited induced fit upon leptin binding, enhancing shape complementarity at the interface (*43*).

We then applied this leptin-neutralizing antibody to a set of well-established mouse models of liver, kidney, and lung fibrosis, namely HFD-fed Mup-uPA transgenic mice, folic acid–treated wild-type mice, and bleomycin-treated wild-type mice, demonstrating that leptin neutralization effectively mitigates fibrosis development. Under the chosen conditions, antibody treatment resulted in a considerable decrease in local ECM deposition and inflammatory gene expression as well as an overall improvement of tissue architecture. We thus not only provide compelling evidence that leptin signaling promotes peripheral tissue fibrosis but also highlight the immense potential of leptin neutralization as a therapeutic strategy to fibrotic diseases.

Leptin has been suggested to contribute to obesity-associated cardiac fibrosis as well (*24*). We therefore also studied the impact of leptin neutralization on the development of cardiac and renal fibrosis in lean, AngII-treated wild-type mice. Notably, while neutralizing leptin had no noticeable effect on the hypertension-induced progression of cardiac fibrosis, it still attenuated renal fibrosis in this model, suggesting that leptin signaling may influence fibrotic processes in a tissue- and/or insult-dependent manner. Future studies should include obese models to further explore the contributions of leptin signaling to the development of cardiac fibrosis and clarify the therapeutic applicability of leptin neutralization to this condition.

Antibody-mediated leptin neutralization had no adverse effects on body weight in any of the fibrotic models we studied. We suppose that the dose of antibody we applied results in a partial, but not full, neutralization of leptin activity so that sufficient central leptin signaling remains to prevent any detrimental effects on energy homeostasis from manifesting (*5*). Our findings thus suggest that leptin activity can be modulated therapeutically in both the obese and lean states without immediately compromising energy homeostasis.

Our study establishes a key role for leptin signaling in the development of peripheral tissue fibrosis. However, whether the antibody-mediated neutralization of leptin we perform here exerts its beneficial effects through changes in central and/or peripheral, potentially local, leptin signaling remains unresolved. LEPRb is not only expressed centrally by different populations of neurons but also peripherally by fibrogenic cells, including hepatic stellate cells, renal interstitial fibroblasts, lung fibroblasts, and cardiac fibroblasts (*44*–*46*), as well as in immune cells, including macrophages and T cells (*47*). As demonstrated by others, the activation of LEPRb and subsequent engagement of JAK2-STAT3, PI3K-AKT, and MAPK pathways in these peripheral cell types can directly promote their profibrotic and pro-inflammatory activation (*44*, *47*). Here, we observed a local increase in the expression of LEPRb, phosphorylation of STAT3, and expression of SOCS3 in the livers of HFD-fed Mup-uPA transgenic mice and kidneys of folic acid–treated wild-type mice, suggesting that there may be a relevant contribution of local leptin signaling in fibrogenic and/or immune cells to the progression of fibrosis in these models. Dedicated loss-of-function studies will be needed to dissect the relative contributions of central and peripheral leptin signaling in distinct cell types to the development of fibrosis in different peripheral tissues.

Previous studies have suggested peripheral leptin signaling to play both direct, profibrotic and indirect, pro-inflammatory roles in the progression of peripheral tissue fibrosis (*48*–*51*). Whether direct or indirect leptin actions predominate may depend on the specific tissue and insult in question. While studies in liver and kidney fibrosis models have suggested direct, profibrotic actions that also implicate transforming growth factor–β (TGF-β) and STAT3 signaling (*50*, *51*), a direct profibrotic action of leptin could not be established in vivo. Leptin has however been shown to enhance the expression of collagen I and α–smooth muscle actin in TGF-β1–stimulated A549 cells in vitro. Concerning indirect, pro-inflammatory effects, leptin has been demonstrated to augment the expression of TNF-α (tumor necrosis factor–α), IL-12, and IL-6 in LPS-stimulated macrophages (*52*).

Although central leptin resistance is a well-recognized phenomenon that has been proposed to contribute to the development and maintenance of obesity by impairing neuronal leptin signaling, the occurrence and relevance of obesity-associated peripheral leptin resistance remain ill-defined. Reports from others, as well as our own findings, suggest that leptin signaling may persist in peripheral tissues in the obese state. Fernández-Riejos *et al.* (*47*), for instance, demonstrated that peripheral leptin signaling remained active and modulated immune responses and tissue remodeling, even in a setting of systemic hyperleptinemia. Similarly, we observed indications of peripheral leptin signaling in the livers of HFD-fed Mup-uPA transgenic mice, specifically increases in local STAT3 phosphorylation and SOCS3 expression. Central leptin resistance has been suggested to arise from an impaired transport of leptin across the blood-brain barrier, diminished expression of LEPRb on the surface of neurons, detrimental posttranslational modifications to LEPRb, and/or negative feedback through SOCS3 and PTP1B (tyrosine-protein phosphatase nonreceptor type 1) (*53*–*55*), the causes of mechanisms underlying the development of peripheral leptin resistance have remained comparably elusive. Inflammatory cytokines may actually enhance the expression of LEPRb and downstream signaling in peripheral tissues, creating a feed-forward loop that may augment fibrosis (*56*, *57*). The relative preservation of leptin sensitivity in peripheral tissues in the context of fibrosis may reflect both a unique tissue microenvironment and specific signaling events.

While our study highlights the therapeutic potential of leptin neutralization, certain limitations must be acknowledged. The dose of leptin-neutralizing antibody may require further optimization across different fibrotic conditions, and the long-term safety of antibody application remains to be explored, although we now anticipate no negative effects. In addition, expanding this research to include female as well as obese mice will enhance the translational relevance of these findings.

In conclusion, our study demonstrates that targeting leptin signaling is a promising strategy for treating fibrosis in multiple organs. Our findings highlight the intersection of metabolic, inflammatory, and fibrotic processes, emphasizing the importance of developing comprehensive therapeutic approaches to chronic diseases. We thus lay the groundwork for future research into the targeted modulation of leptin signaling and its clinical applications.

## MATERIALS AND METHODS

### Study design

The aim of this study is to define the structural and therapeutic potential of a leptin-neutralizing antibody (hLep3) in tissue fibrosis. Structural characterization was performed using x-ray crystallography to solve the structures of hLep3 in both unbound and leptin-bound states, revealing that the antibody mimics the LEPR by engaging key leptin-binding interfaces. To assess therapeutic efficacy, hLep3 was tested in multiple murine models of fibrosis, affecting the kidney, liver, lung, heart, and vasculature. Mice received hLep3 or IgG control, and fibrosis was evaluated using histology, immunostaining, and gene expression. Mechanistic studies further examined the impact of leptin blockade on inflammatory and fibrotic signaling pathways including JAK-STAT. Only male mice were used in this study, and treatments included folic acid, bleomycin, and AngII with or without hLep3. In each experiment, age-matched male mice were randomly assigned to treatment groups. Mup-uPA mice were fed an HFD followed by IgG- or LepAb-neutralizing antibody injection, and the body weight was monitored. Investigators were not blinded to genotyping or treatment groups at Touchstone Diabetes Center, Dallas, TX. Mice were maintained, and studies were performed according to protocols approved by the Institutional Animal Care and Use Committee of University of Texas Southwestern Medical Center. Mice had ad libitum access to standard or special diets, and all experiments were replicated at least once to ensure biological reproducibility and adequate statistical analysis for comparisons.

### Animal models

The Institutional Animal Care and Use Committee of University of Texas Southwestern Medical Center at Dallas approved all animal studies (APN no. 2024-103545-G). All mice were housed under standard laboratory conditions (12-hour light/12-hour dark cycle) and provided with ad libitum access to food and water.

### Transgene and HFD-induced liver fibrosis model

Twelve-week-old male Mup-uPA transgenic mice (*58*) and their transgene-negative littermates were fed a lard-based HFD (60% calories from fat; Bio-Serv, no. S1850) for 10 weeks to promote obesity and then grouped to receive either LepAb or control IgG injections for another 2 weeks. These injections were performed intraperitoneally, twice per week at a dose of 10 mg/kg. The mice were euthanized after 2 weeks of antibody injection, and tissues were collected for analysis.

### Folic acid–induced kidney fibrosis model

Ten- to 12-week-old male C57Bl/6 mice were injected intraperitoneally with a single dose of folic acid (200 mg/kg in 0.3 M NaHCO_3_, pH 7.4; Sigma-Aldrich, F7876) or vehicle (0.3 M NaHCO_3_, pH 7.4) to induce acute kidney injury and subsequent fibrosis and then grouped to receive either LepAb or control IgG injections for 21 days, starting on day 1. These injections were performed intraperitoneally, every other day at a dose of 1 to 20 mg/kg (as indicated). The mice were euthanized at day 28, and tissues were collected for analysis.

### Bleomycin-induced lung fibrosis model

Twelve-week-old male C57Bl/6 mice were administered intratracheally with a single dose of bleomycin (1.5 units/kg; Sigma-Aldrich, no. B2434) or vehicle (saline) to induce acute lung injury and subsequent fibrosis and then grouped to receive either LepAb or control IgG injections for 14 days, starting on day 1. These injections were performed intraperitoneally, every other day at a dose of 10 mg/kg. The mice were euthanized at day 14, and tissues were collected for analysis. Another group of mice was not treated with any antibody and euthanized at day 14.

### BP measurement

Minipumps (1002D; Alzet, Cupertino, CA) were implanted subcutaneously in 11- to 12-week-old mice to deliver AngII (1000 ng/kg per minute) or vehicle (saline) (*59*). For tail-cuff BP measurements, mice were infused with AngII or vehicle for 2 weeks, and BP was measured using the CODA-HT8 Blood Pressure Analysis System (CODA High Throughput system**,** Kent Scientific Corp., Torrington, CT).

### Serum BUN measurement

The levels of serum BUN were measured using an Invitrogen Urea Nitrogen Colorimetric Detection kit (Invitrogen, EIABUN).

### Serum creatinine measurement

The levels of serum creatinine were measured using a Cayman Creatinine (serum) Colorimetric Assay kit (Cayman, no. 700460).

### Serum AST and ALT measurement

The measurement of serum AST and ALT was performed by the Metabolic Phenotyping Core of UTSW Medical Center.

### HEK293 cell culture, transfection, and luciferase assay

HEK293 cells were cultured in Dulbecco’s modified Eagle’s medium (Gibco, 11965092) supplemented with 10% fetal bovine serum and penicillin-streptomycin in a humid incubator with 5% CO_2_ at 37°C. This cell line was seeded into a 96-well plate and reached 80% confluence. The cells were then cotransfected with LEPRb and p-STAT3:luciferase reporter plasmids and treated with leptin (40 ng/ml) in the presence of either LepAb or a control IgG antibody. Before adding to the wells, leptin and LepAbs were mixed in 1.5-ml Eppendorf tubes with gentle shaking for 1 hour. Then, the mixture was added into the well for 24 hours. After that, the wells were washed twice with cold phosphate-buffered saline and then underwent luciferase analysis using the ONE-Glo Luciferase Assay System (*60*).

### Leptin-neutralizing antibody discovery and production

The generation and purification of the human leptin-neutralizing antibody (hLep3; herein “LepAb”) are described in detail elsewhere (*5*).

### Human leptin production

A pSUMO-TCS(A)-LEP W100E plasmid for human leptin [W100E variant (*61*)] expression in bacterial cells was cloned by Gibson assembly. This plasmid encodes a fusion protein featuring an N-terminal hexahistidine-affinity tag, SUMO, a tobacco etch virus (TEV) protease-cleavable linker, and mature human leptin (amino acids 22 to 167; with W121E substitution). The pSUMO-TCS(A)-LEP W100E plasmid was transformed into the expression host *Escherichia coli* BL21 (DE3), and overexpression was induced overnight at 30°C. The cells were lysed, and Ni-NTA purification was performed. This yielded a recombinant protein of ~95% purity by SDS-PAGE. The hexahistidine-affinity tag and SUMO were subsequently removed by TEV protease digestion. Ni-NTA extraction of the removed portions and used TEV protease resulted in a recombinant protein of ~98% purity by SDS-PAGE (Fig. 1A). The pSUMO-TCS(A)-LEP W100E plasmid sequence is available upon request.

### Fab generation and Fab:leptin complex formation

LepAb (25 mg/ml) was digested with agarose-immobilized papain (Thermo Fisher Scientific, no. 20341) to produce Fab and Fc antibody fragments. For complex formation, the purified Fab (12 mg/ml) was incubated with recombinant human leptin (15 mg/ml) overnight at 4°C. An HL Sephacryl S-200 HR column (Cytiva, no. 17119501) was used for size exclusion chromatography using a 20 mM tris-HCl (pH 7.5) and 150 mM NaCl buffer. Peak fractions containing the desired protein or protein complexes were pooled and concentrated to 20 mg/ml using a Pierce 10K MWCO Protein Concentrator (Thermo Fisher Scientific, no. 88528).

### Crystallization, data collection, and structure determination

The Fab:leptin complex and unbound Fab crystals were grown by the hanging drop vapor diffusion method at 20°C in 24-well VDX trays using a 1:1 ratio of protein/reservoir solution. The Fab:leptin complex was applied at 25 mg/ml in a buffer containing 20 mM Hepes, pH 7.4, and 75 mM NaCl against a reservoir solution containing 18% polyethylene glycol, molecular weight 800 (PEG-8000) and 20% glycerol. The unbound Fab was applied to VDX trays at 25 mg/ml in a buffer containing 20 mM Hepes, pH 7.4, and 75 mM NaCl against a reservoir solution containing 1 M LiCl, 0.1 M citrate, pH 4.0, and 20% (w/v) PEG-8000. The obtained crystals were cryoprotected with 18% (w/v) PEG-8000 and 20% (v/v) glycerol, diffracted to a minimum Bragg spacing (*d*_min_) of 3.10 Å, and exhibited the symmetry of space group *P*1 with cell dimensions of *a* = 51.9 Å, *b* = 48.5 Å, *c* = 125.0 Å, α = 90.3°, β = 85.2°, and γ = 70.4° and contained two complexes per asymmetric unit. Crystals of the unbound Fab diffracted to a minimum Bragg spacing (*d*_min_) of 3.25 Å and exhibited the symmetry of space group *P*2_1_2_1_2 with cell dimensions of *a* = 70.2 Å, *b* = 207.9 Å, and *c* = 72.8 Å and contained two molecules of Fab per asymmetric unit. All diffraction data were collected at beamline 19-ID (SBC-CAT) at the Advanced Photon Source (Argonne National Laboratory, Argonne, IL) and processed in the program HKL-3000 (*62*) with applied corrections for effects resulting from absorption in a crystal and for radiation damage (*63*, *64*), the calculation of an optimal error model, and corrections to compensate the phasing signal for a radiation-induced increase in nonisomorphism within the crystal (*65*, *66*). The data for both crystals displayed notable levels of anisotropy.

Phases were obtained via a molecular replacement experiment in the program Phaser (*67*) using modified search models from the previously determined human leptin structure (PDB: 1AX8) and a model for the heavy and light chains of the Fab obtained from the AlphaFold2 CoLab server (*68*, *69*). Completion of this model was performed by multiple cycles of manual rebuilding in the program Coot (*70*) alternated with positional and isotropic atomic displacement parameter refinement to a resolution of 3.10 Å using the program Phenix (*71*), with a random 10% of all datasets aside for an *R*_free_ calculation. Because of the low resolution of the diffraction data for both crystals, torsion-angle noncrystallographic symmetry restraints and secondary structure restraints were used during model refinement. The model and electron density for chains A (Fab heavy chain), B (Fab light chain), and C (leptin) of the Leptin:Fab complex are the most complete. The more complete model of leptin in this complex includes an ordered helix E and the linker between helices D and E, primarily due to a close crystallographic lattice contact to this ordered region. Data collection and structure refinement statistics are summarized in table S1.

### RNA isolation and RT-qPCR

Tissue samples were lysed at 4°C in TRIzol (Thermo Fisher Scientific, no. 15596018), and RNA was isolated using the RNeasy Mini Kit (Qiagen, no. 74106). RNA concentrations were determined on a NanoPhotometer (Implen), and cDNA synthesis was carried out using the PrimeScript 1st strand cDNA Synthesis Kit (Takara, no. 6110A). RT-qPCR was performed using PowerUp SYBR Green Master Mix (Thermo Fisher Scientific, no. A25778) on the QuantStudio 6 Flex Real-Time PCR System (Thermo Fisher Scientific). Gene expression was normalized to the housekeeping gene *Rpl19* using the ΔΔ*C*_T_ method. PCR specificity was confirmed by the melting curve analysis. Primer sequences are listed in table S2.

### RNA-seq analyses

RNA-seq was performed by Novogene (Sacramento, CA), as described previously (*72*). Briefly, RNA isolated from the kidney or lung was used to prepare an RNA-seq library. Downstream analysis was done from the matrix file provided by Novogene. For each comparison, differentially expressed genes were identified using a threshold of log_2_ fold change >0.5 and <−0.5 and an adjusted *P* value (*P*_adj_) <0.05. GO enrichment analysis was carried out separately for the up-regulated and down-regulated genes using the cluster Profiler package in R focusing on the Biological Process category. All expressed genes in the dataset were used as the background, and only GO terms with a *q* value <0.05 were considered significant along with the gene count of >=10. Across the comparisons, we observed different sets of enriched pathways depending on the direction of gene regulation. Enrichment of pathways affected in both directions was plotted in each comparison on the basis of the statistical significance and gene count.

### Protein extraction and immunoblot analysis

Tissues were lysed at 4°C in radioimmunoprecipitation assay (Sigma-Aldrich, R0278) buffer supplied with protease and phosphatase inhibitors (Roche, no. 04906837001) and subsequently cleared by centrifugation. Protein concentrations were determined using the Pierce BCA Protein Assay (Thermo Fisher Scientific, no. 23225). Proteins were separated into two gels (Bio-Rad, no. 5671095) and transferred onto two membranes (Bio-Rad, no. 1704271). The following primary antibodies were used at 1:1000 dilutions: p-STAT3 (Cell Signaling Technology, no. 4139), STAT3 (Sigma-Aldrich, no. A4700), and SOCS3 (Cell Signaling Technology, no. 52113). Membranes were incubated with IRDye-conjugated secondary antibodies (LI-COR) and scanned on an Odyssey DLx Imager (LI-COR). The obtained images were analyzed using Image Studio software (version 3.0; LI-COR).

### Histological analysis

Tissues were fixed overnight at room temperature in 10% neutral-buffered formalin and thereafter stored in 50% ethanol. Fixed tissues were dehydrated, embedded in paraffin, and cut into 4- to 7-μm sections. H&E, picrosirius red, and Masson’s trichrome staining of deparaffinated sections was carried out according to established protocols. For immunofluorescence staining, deparaffinated sections were treated for 20 min at 95°C in R-buffer A (Electron Microscopy Sciences, 62706-10) for antigen retrieval, blocked with 5% normal goat serum for 60 min at 22°C, incubated with primary antibodies overnight at 4°C, incubated with fluorochrome-conjugated secondary antibodies at 37°C for 1 hour, and then mounted with antifade mounting medium (Vectashield). The following primary antibodies were used: fibronectin (1:500 dilution; Chemicon, no. AB1943), collagen I (1:500 dilution; Thermo Fisher Scientific, no. PA5-95137), and R-CHP (1:500 dilution; Advanced Biomatrix, no. 5276-UG). Samples were imaged on a Zeiss Axioscan 7.

### Statistical analysis

The data are expressed as the means ± SEM. Differences between groups were assessed using an analysis of variance (ANOVA). Statistical significance was determined at *P* < 0.05 using either a one-tailed or two-tailed Student’s *t* test or one-way ANOVA with Bonferroni’s post hoc test, as appropriate. Detailed statistical information, including the exact sample size (*n*), measures of central tendency, dispersion, precision (means ± SEM), and significance levels, is provided in the figures and figure legends. All statistical analyses were conducted with GraphPad Prism 10.4.1.
