# Supplementary material for: Leptin as a key driver for organ fibrogenesis
Source: Sci Adv. 2025 Oct 22;11(43):eady7904. doi: 10.1126/sciadv.ady7904 (PMC12542948; doi:10.1126/sciadv.ady7904)
Supplement: Supplementary file 1 — Figs. S1 to S4 Tables S1 and S2 Legend for table S3 [file sciadv.ady7904_sm.pdf]

Science Advances
AAAS

Supplementary Materials for

Leptin as a key driver for organ fibrogenesis

Xue-Nan Sun *et al.*

Corresponding author: Philipp E. Scherer, philipp.scherer@utsouthwestern.edu

Sci. Adv. **11**, eady7904 (2025)
DOI: 10.1126/sciadv.ady7904

The PDF file includes:

Figs. S1 to S4
Tables S1 and S2
Legend for table S3

Other Supplementary Material for this manuscript includes the following:

Table S3

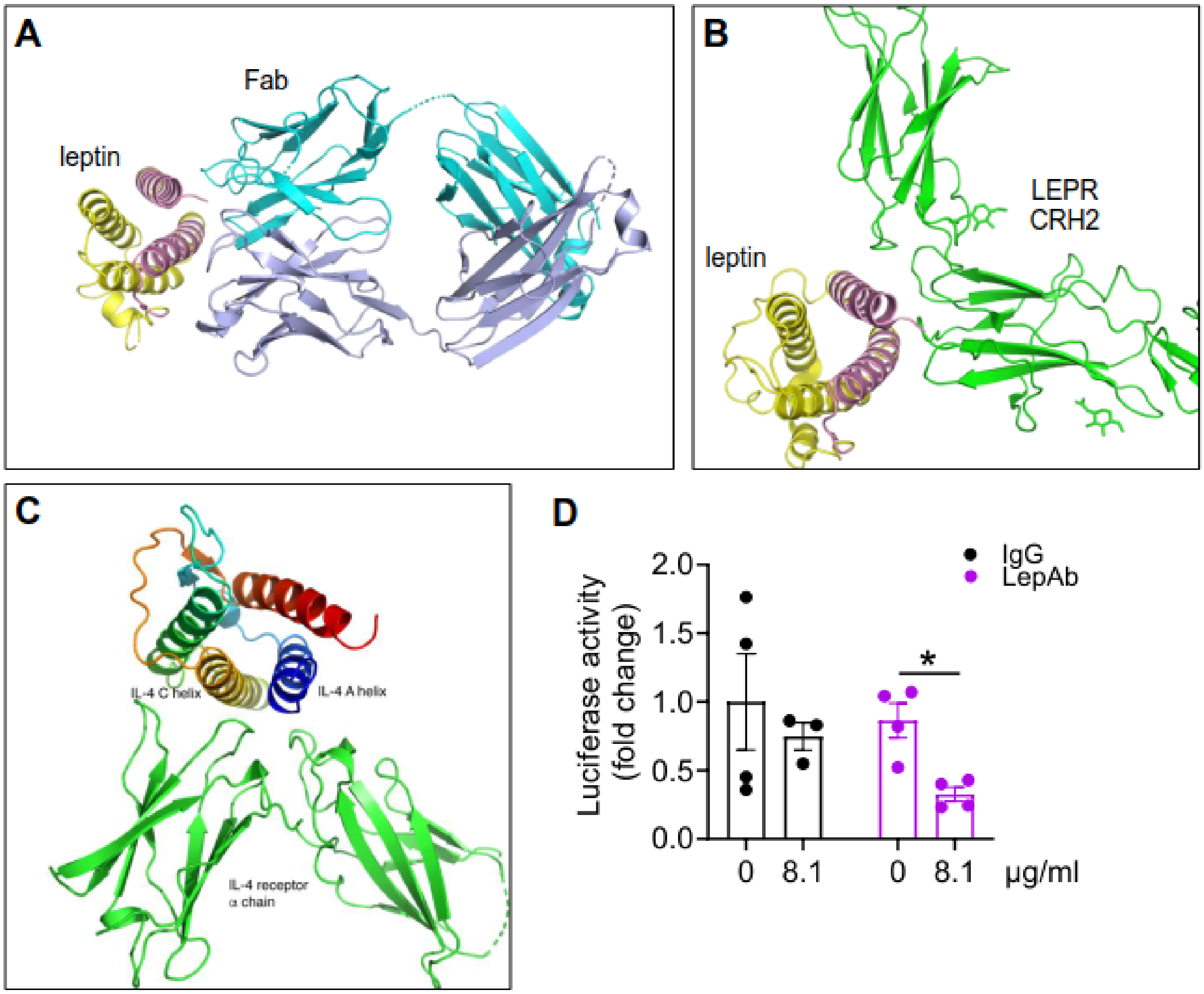

Fig. S1. Further characterization of a leptin-neutralizing antibody.

(A) Overview of the Fab: leptin complex structure. Leptin (colored yellow and with helices A and C in pink) is shown docked with the Fab light chain (colored cyan) and heavy chain (colored light blue). **(B)** Overview of the leptin receptor (LEPR) CRH2: leptin complex. Leptin (colored yellow and with helices A and C in pink) is shown docked with the CRH2 domain (colored green; PDB: 8X80 (59)). **(C)** Overview of the interleukin-4 (IL-4) receptor α chain:IL-4 complex. IL-4 (multicolored) is shown docked with the IL-4R α chain (colored green; PDB: 1IAR). This interaction closely resembles the extended interaction of the leptin-neutralizing Fab with leptin. Buried surface areas are 810 Å2 for the IL-4R α chain: IL-4 complex (26), 760 Å for the LEPR CRH2: leptin complex, and 870 Å2 for the leptin-neutralizing Fab: leptin complex. The latter two according to the Protein Interfaces, Surfaces and Assemblies (PISA) service at the European Bioinformatics Institute. (http://www.ebi.ac.uk/pdbe/prot_int/pistart.html) (60). **(D)** pSTAT3 response in HEK293 cells co-transfected with LEPRb and a pSTAT3: luciferase reporter plasmids and

stimulated for 12 hours with leptin (40 ng/ml) in presence or absence of either LepAb or control IgG (n=3-4 per group). Data are presented as mean±SEM and were analyzed by a two-tailed Student's t test. *, p< 0.05.

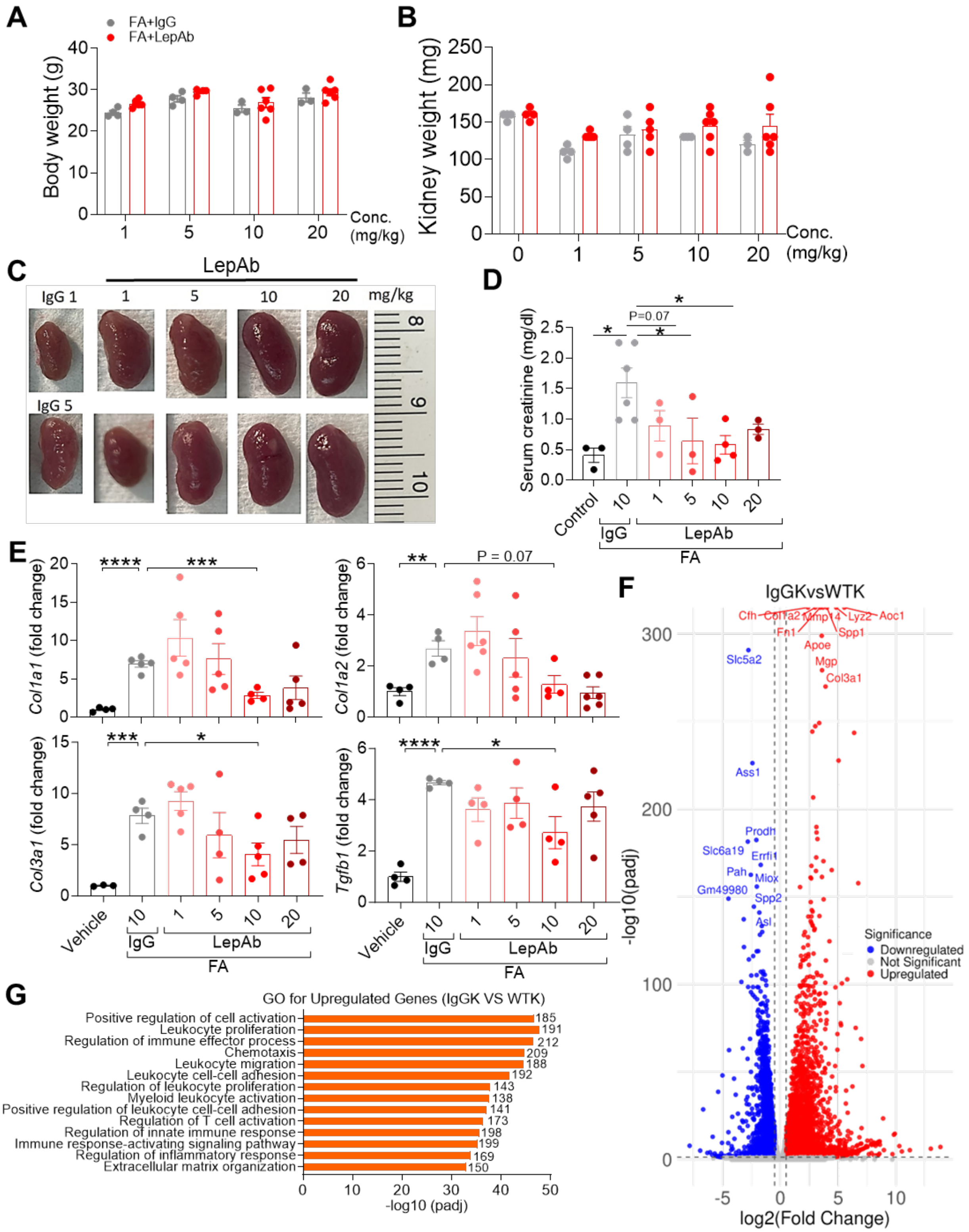

Fig. S2. Further effects of leptin neutralization in kidney fibrosis.

Refer to Figure 3 for experimental setup. **(A−B)** Body and kidney weight following folic acid (FA)-induced kidney fibrosis (n=3-6 per group). **(C)** Gross anatomy of kidney (n=1-2 per group). **(D)** Serum blood creatinine levels. **(E)** RT-qPCR analysis of fibrotic gene mRNA expression in the kidney (n=3-6 per group) in does-dependant experiments followed by FA-induced kidney fibrosis. **(F)**. Volcano plots display fold change (x-axis) versus adjusted *P* value (y-axis) of transcriptomic data from kidneys of vehicle-treated (WTK) vs. folic acid–treated (IgGK) mice. Mean values from n = 3 / group. **(G)**. Labels identify gene clusters showing enrichment GO analyses for Kid-IgG-(IgGK) and Kid-Vehicle (WTK) treated mice.
(A−B) Data are presented as mean±SEM and were analyzed by a one-way ANOVA test. No significant differences were detected.

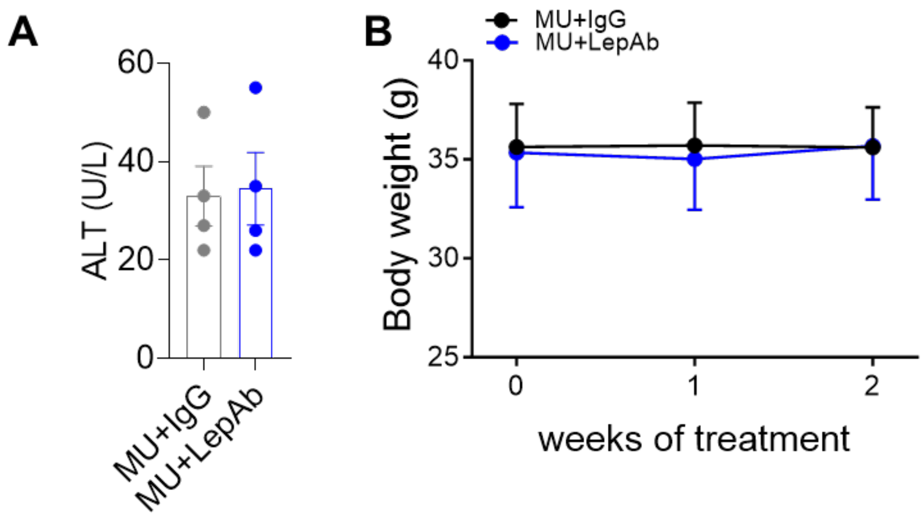

Fig. S3. Additional effects of leptin neutralization in liver fibrosis.
Refer to Figure 4 for experimental setup. **(A)** Serum alanine aminotransferase (ALT) levels. **(B)** Body weight (n=7 per group). Data are presented as mean±SEM and were analyzed by one-way ANOVA. No significant differences were detected.

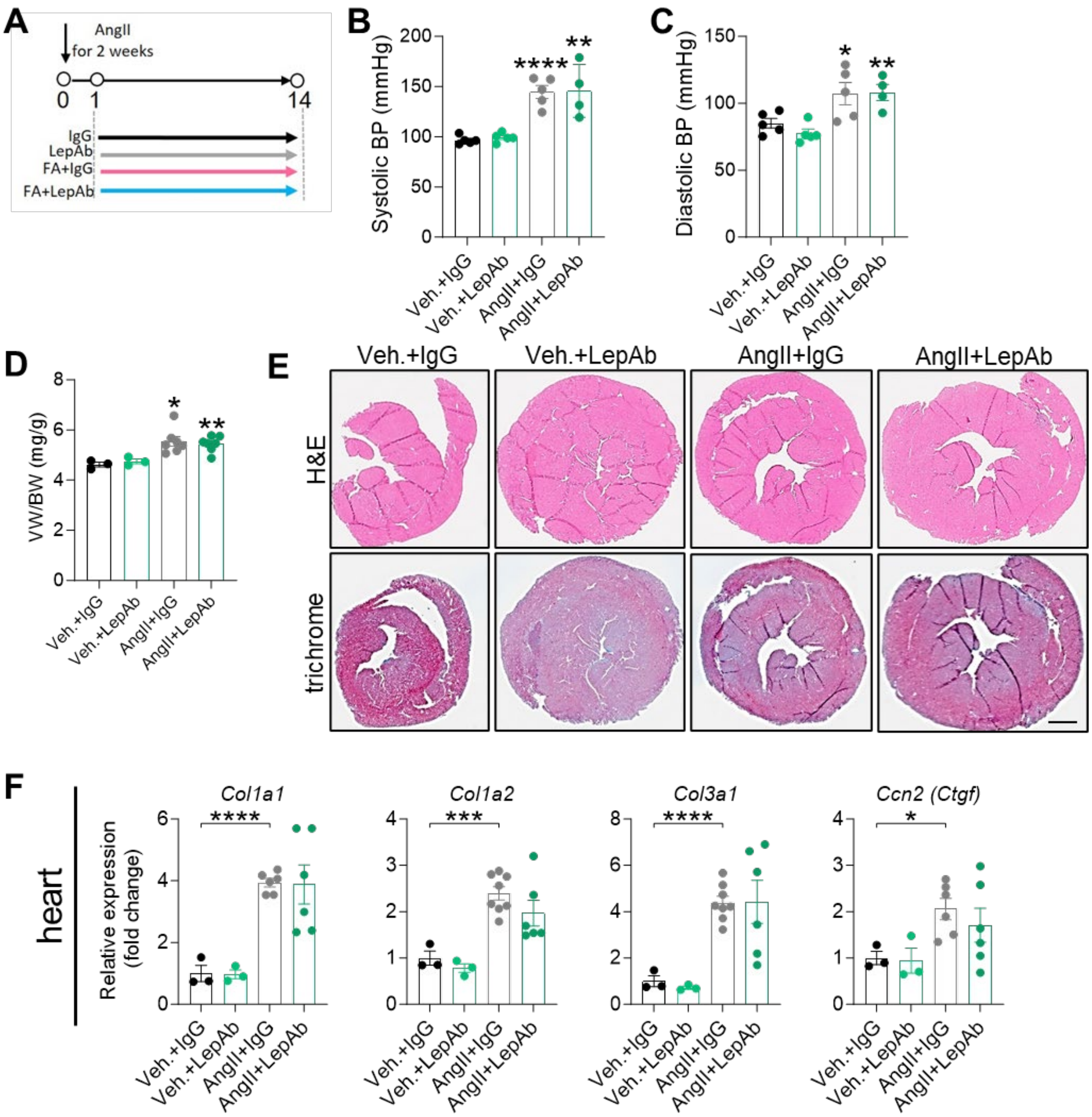

Fig. S4. Leptin neutralization mitigates AngII-induced aorta and kidney fibrosis, but not heart fibrosis.

(A) Experimental setup of angiotensin (Ang II)-induced heart and kidney fibrosis. **(B−C)** Tail-cuff measurements of systolic **(B)** and diastolic **(C)** blood pressure (n=4-5 per group). **(D)** Ratio of ventricular weight to body weight (n=3-6 per group). **(E)** H&E and Masson's trichome staining of heart. Representative microphotographs are shown (n=4 per group). Scale bar equals 1mm. **(F)** RT-qPCR analysis of fibrotic gene mRNA expression in the heart (n=3-6 per group). (Scale bar equals 50 µm. (B-D, F-G, I, and K) Data are presented as mean±SEM and were analyzed by one-way ANOVA. *, $p < 0.05$; **, $p < 0.01$; ***, $p < 0.001$; ****, $p < 0.0001$. H&E, hemoxylin and eosin.

Table S1. Data collection and refinement statistics, Leptin: Fab and Fab structures.

**Data collection**		
Crystal	Leptin: Fab	Fab
Space group	P1	$P2_12_12$
Cell constants (Å, °)	a = 51.93, b = 48.53, c = 124.96, α = 90.34, β = 85.21, γ = 70.42	a = 70.22, b = 207.94, c = 72.82
Wavelength (Å)	0.97918 (2/17/2023, APS-173)	
Resolution range (Å)	40.17 – 3.00 (3.19 – 3.00)	49.33 – 3.25 (3.31 – 3.25)
Unique reflections	20,607 (820)	17,545 (871)
Multiplicity	1.8 (1.4)	21.4 (7.6)
Data completeness (%)	88.9 (66.5)	100.0 (99.9)
R_{merge} (%)[a]	20.0 (122.7)	16.4 (143.9)
R_{pim} (%)[b]	13.5 (84.9)	3.60 (52.4)
I/σ(I)	5.4 (0.9)	23 (1.9)
$CC_{1/2}$ (highest resolution shell)	0.80	0.68
Wilson B-value (Å^2)	59.2	47.5
**Phase determination**		
MR models (structure)	PDB ID 1AX8 (leptin), AlphaFold2 model[c] (Fab)	Leptin: Fab (Fab)

**Refinement statistics**		
Crystal	Leptin: Fab	Fab
Resolution range (Å)	40.17 – 3.10 (3.19 – 3.10)	49.33 – 3.30 (3.41 – 3.30)
No. of reflections R_{work}/R_{free}	16,569/1,648 (629/85)	15,683 (1,576)
Data completeness (%)	79.8 (41.0)	93.9 (45.0)
Atoms (non-H protein/ions)	8,142/10	6,240
R_{work} (%)	23.6 (29.2)	26.3 (30.0)
R_{free} (%)	27.7 (39.4)	32.1 (36.1)
R.m.s.d. bond length (Å)	0.003	0.002
R.m.s.d. bond angle (°)	0.57	0.55
Mean B-value (Å^2) (chains A/B/C/D/E/F/ions)	49.8/52.0/60.6/52.0/56.0/63.4/62.1	55.8/68.1/64.3/53.6
Ramachandran plot (%) (favored/additional/disallowed)[d]	93.9/5.7/0.4	95.4/4.6/0.0
Clashscore/Molprobity[d] overall score	1.1/1.4	7.1/1.4
Maximum likelihood coordinate error	0.47	0.50

Missing residues	A: 1, 136-142, 223-225. B: 58-59, 112-113, 214-217. C: 26-47, 107-108. D: 26-28, 136-141, 223-225. E: 116-118, 216-217. F: 26-48, 107-117.	A: 1, 102-107, 136-142, 223-225. B: 1-2, 58-59, 112-113, 214-217. C: 1, 101-105, 223-225. D: 1, 112-113, 214-217.

Data for the outermost shell are given in parentheses.

[a]$R_{\text{merge}} = 100\ \Sigma_h\Sigma_i|I_{h,i} - \langle I_h\rangle|/\Sigma_h\Sigma_i\ \langle I_{h,i}\rangle$, where the outer sum (h) is over the unique reflections and the inner sum (i) is over the set of independent observations of each unique reflection.

[b]$R_{\text{pim}} = 100\ \Sigma_h\Sigma_i\ [1/(n_h - 1)]^{1/2}|I_{h,i} - \langle I_h\rangle|/\Sigma_h\Sigma_i\ \langle I_{h,i}\rangle$, where n_h is the number of observations of reflections **h**.

[c]As generated in the AlphaFold2 ColabFold server(*72, 73*).

[d]As defined by the validation suite MolProbity(*76*).

Table S2. Primer sequences for qPCR

Gene	Primer forward (5' → 3')	Primer reverse (5' → 3')
Ifng	ATGAACGCTACACACTGCATC	CCATCCTTTTGCCAGTTCCTC
Il1b	GCAACTGTTCCTGAACTCAACT	ATCTTTTGGGGTCCGTCAACT
Il6	CCGGAGAGGAGACTTCACAG	CAGAATTGCCATTGCACAAC
Mcp1	TTAAAAACCTGGATCGGAACCAA	GCATTAGCTTCAGATTTACGGGT
Ccn2(Ctgf)	GGGCCTCTTCTGCGATTTC	ATCCAGGCAAGTGCATTGGTA
Lepr	GGTCACCCAGCACAATCCAA	TTGGGCTCAGACGTAGGATG
Leprb	GACGATGTTCCAAACCCCAAG	GAAATGGGTTCAGGCTCCAG
Col1a1	GTGCTCCTGGTATTGCTGGT	AAGGACCATCCCACTGTCTG
Col1a2	GTAACTTCGTGCCTAGCAACA	CCTTTGTCAGAATACTGAGCAGC
Col3a1	ACGTAGATGAATTGGGATCAG	GGGTTGGGGCAGTCTAGTG
Tgfb1	CCTGCAAGACCATCGACATG	TGTTGTACAAAGCGAGCACC
RPL19	ATGAGTATGCTCAGGCTACAGA	GCATTGGCGATTTCATTGGTC
Acta1	CCCAAAGCTAACCGGGAGAAG	CCAGAATCCAACACGATGCC

Table S3. Values for all data points in graphs.
